# Risk factors for chronic obstructive pulmonary disease in never‐smokers: A systematic review

**DOI:** 10.1111/crj.13479

**Published:** 2022-02-10

**Authors:** Ana Pando‐Sandoval, Alberto Ruano‐Ravina, Cristina Candal‐Pedreira, Carlota Rodríguez‐García, Cristina Represas‐Represas, Rafael Golpe, Alberto Fernández‐Villar, Mónica Pérez‐Ríos

**Affiliations:** ^1^ Department of Pneumology Central University Teaching Hospital of Asturias Oviedo Spain; ^2^ Department of Preventive Medicine and Public Health University of Santiago de Compostela Santiago de Compostela Spain; ^3^ Consortium for Biomedical Research in Epidemiology and Public Health (CIBER en Epidemiología y Salud Pública‐ CIBERESP) Madrid Spain; ^4^ Health Research Institute of Santiago de Compostela (Instituto de Investigación Sanitaria de Santiago de Compostela ‐ IDIS) Santiago de Compostela Spain; ^5^ Department of Pneumology University Clinical Teaching Hospital of Santiago de Compostela Santiago de Compostela Spain; ^6^ Department of Pneumology Alvaro Cunqueiro University Teaching Hospital, NeumoVigo I+i Research Group, Southern Galician Institute of Health Research (Instituto de Investigación Sanitaria Galicia Sur ‐ IISGS) Vigo Spain; ^7^ Department of Pneumology Lucus Augusti University Teaching Hospital Lugo Spain; ^8^ Grupo C039 Biodiscovery HULA‐USC Health Research Institute of Santiago de Compostela Santiago de Compostela Spain

**Keywords:** COPD, epidemiological studies, never‐smoker, systematic review

## Abstract

**Introduction:**

Relatively little is known about the risk factors for chronic obstructive pulmonary disease (COPD) in never‐smokers, and these factors have not yet been fully characterised. This study therefore sought to analyse COPD risk factors in never‐smokers by conducting a systematic review of the literature on the topic.

**Materials and methods:**

We performed a search in PubMed (Medline) and Embase from 2000 onwards, to locate studies on COPD in never‐smokers. For literature search and evidence synthesis purposes, we used the PRISMA guidelines and drew up a specific quality scale to quantify the evidence of each study included.

**Results:**

The bibliographic search retrieved a total of 557 papers, 20 of which fulfilled the designated inclusion criteria (two case–control studies, four cohort studies and 14 cross‐sectional studies). These studies were undertaken in Europe, the United States, Latin America, Asia and Africa. The risk factors for never‐smokers were varied and ranged from exposure to biomass, occupational exposure and passive smoking to having a history of asthma, tuberculosis or respiratory infections during childhood. The effect of residential radon was unclear. The highest risk was obtained for previous respiratory diseases of any type, with a magnitude much higher than that observed for other risk factors.

**Conclusions:**

There are few studies on COPD risk factors in never‐smokers. More purpose‐designed studies in this subpopulation are thus called for, including well‐designed studies to specifically assess if indoor radon has any role on COPD onset.

## Graphical abstract

A sizeable proportion of COPD patients are never‐smokers and many risk factors are implicated in the disease's development, fundamentally exposure to biomass fuel, risk occupations, a history of asthma or tuberculosis, and exposure to passive smoking. The effect of residential radon was unclear. More studies in this subpopulation are thus called for, including indoor radon measurements.

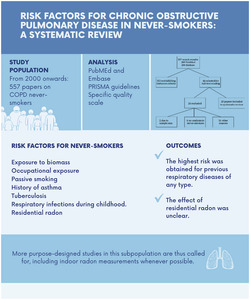

## 1 INTRODUCTION

Chronic obstructive pulmonary disease (COPD) is defined as a preventable and treatable disease, characterised by persistence of respiratory symptoms and limitation of airflow due to abnormalities of the respiratory tract and/or alveolar duct, generally caused by exposure to toxic gases or mediated by occupational exposure or of some other kind.^1^ It is a disease with a high morbidity and mortality worldwide^2^ and ranks as the third leading cause of death in the world.^3^ According to the results of the EPISCAN II (Epidemiologic Study of COPD in Spain) study, its prevalence in Spain among the population aged 40 years and over is 11.8% (14.6% in men and 9.4% in women).^4^

Smoking has been identified as the principal risk factor for development of COPD.^5^ That said, however, 25%–45% of all patients with COPD are never‐smokers,^6^ though this prevalence varies significantly depending on the geographical area and on the different epidemiological studies. In Spain, data from the EPISCAN II study show that 27% of patients with COPD are never‐smokers.^4^ Yet despite this relatively high incidence of COPD in never‐smokers, few studies have exclusively targeted these subjects, and there is a great degree of ignorance about the specific effect of various risk factors that may have an influence on the appearance of COPD in this subpopulation and the magnitude of such effect. A recent review has indicated that the study of risk factors in never‐smokers is a challenge.^7^ Chief among factors other than tobacco associated with a higher risk of COPD are genetic and environmental factors. Among environmental risk factors associated with development of COPD, mention has been made of exposure to biomass fumes, occupational exposure to dust and fumes (in agriculture, animal husbandry, mining, construction, exposure to chemical products in industry), environmental pollution, exposure to passive smoking, chronic asthma and tuberculosis.^8^, ^9^ Furthermore, there is evidence to show that exposure to residential radon may also be associated with COPD mortality.^10^

The high incidence and prevalence of COPD, coupled with the existence of risk factors other than tobacco, make it pertinent to carry out a review and synthesis of existing studies that have analysed the risk factors for development of COPD in never‐smokers, by means of conducting a systematic review of the scientific literature.

## 2 METHODS

### 2.1 Data sources and search strategy

A literature search was undertaken in PubMed (Medline) and Embase. The search period covered the preceding 20 years (using ‘publication since 2000’ as the filter), with the last search being made on 1 January 2021. The search was conducted as follows: using the free‐text terms, ‘COPD and (never‐ or non‐smokers)’; applying language filters (English and Spanish); and excluding all communications to congresses, editorials and monographs.

For the literature search and evidence synthesis purposes, we used the Preferred Reporting Items for Systematic Reviews and Meta‐Analyses (PRISMA) guidelines.^11^

### 2.2 Inclusion and exclusion criteria

The following inclusion and exclusion criteria were applied to select studies for the systematic review: (a) Study designs could range from cross‐sectional, case–control and cohort studies to systematic reviews and meta‐analyses; (b) to be eligible, studies had to be on human beings, whether in the general population or in a hospital setting; (c) risk factors analysed had to include occupation, passive smoking, infections in childhood, exposure to biomass in cooking/heating, history of tuberculosis, chronic asthma and exposure to residential radon; (d) studies included had to have a minimum of 100 participants, at least 20 of whom had to be never‐smoker patients with COPD; (e) studies that failed to specify the results in never‐smokers were excluded; (f) diagnosis of COPD in patients in the studies included had to be based on self‐reported patient symptoms or spirometric criteria consisting of pre‐bronchodilator values of FEV1/FVC < 70% or post‐bronchodilator values of FEV1/FVC < 70%.

A ‘never‐smoker’ was defined a person who met at least one of the following conditions: anyone who has smoked (a) fewer than 100 cigarettes in his/her lifetime or (b) less than one cigarette per day during a period of no less than 6 months.^12^ In any case where this definition did not exist, non‐smokers in the studies included had to be referred to as a ‘never‐smoker’ rather than a ‘non‐smoker’.

### 2.3 Extraction and synthesis of data from the studies included

Data were extracted homogeneously from the studies included using a purpose‐designed data‐extraction table that listed the author(s), year of publication, sample size, study design, risk factors analysed, results obtained and study quality. We were unable to perform a meta‐analysis due to the high heterogeneity of the studies included. The information on each study is shown in an evidence table, along with a final qualitative conclusion.

### 2.4 Quality assessment of the studies included

To assess study quality, we drew up a quality scale made up of the following five items: sample size; number of COPD cases in never‐smokers; results adjusted for covariates; study design; and diagnosis of COPD. Each item has a different score, which makes it possible to rate study quality on a scale from 0 to 10 points, with 10 being the maximum score. The scale is shown in Table 1.

**TABLE 1:** Quality scale used to assess the included studies

Item assessed	Characteristic	Weight
Total sample size	100–1000	0
	1001‐10 000	1
	>10 000	2
	
Number of COPD never‐smoker cases	20–100	0
	101–300	1
	>300	2
Covariate adjustment (number)	2 (age and sex)	0
	>2	2
Study design	Cross‐sectional study	0
	Case–control study	1
	Cohort study	2
COPD diagnosis	Spirometry not registered or patient‐reported symptoms	0
	Pre‐bronchodilator FEV1/FVC < 70%	1
	Post‐bronchodilator FEV1/FVC < 70%	2
Total		10

## 3 RESULTS

### 3.1 Search results

**FIGURE 1:**
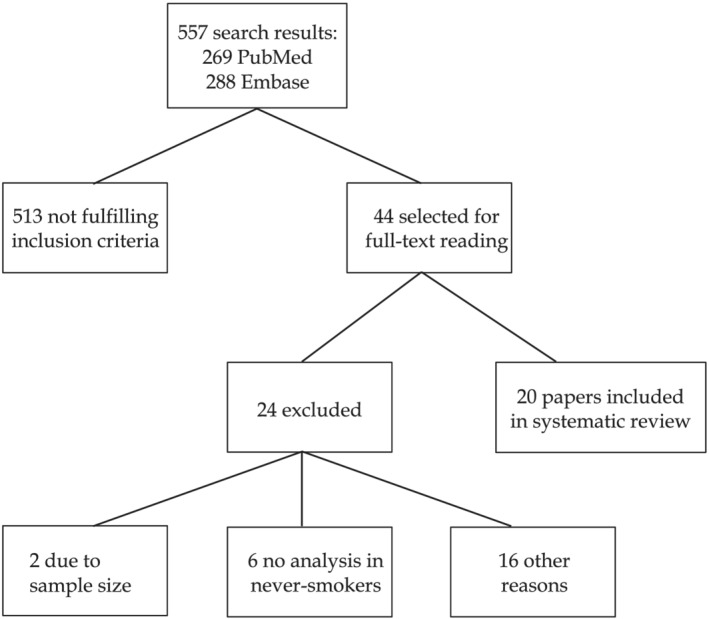
Flow chart showing the inclusion and exclusion process

A total of 557 studies were retrieved from the bibliographic search in PubMed and Embase. After perusal of the abstracts, 44 studies were selected for a full‐text reading. Of these, 20 finally met the established inclusion criteria and comprised two case–control studies, four cohort studies and 14 cross‐sectional studies. The studies were undertaken in Europe, the United States, Latin America, Asia and Africa. The most frequent exclusion criteria were sample size of fewer than 20 cases of COPD in never‐smokers and the fact that the analysis of results failed to differentiate never‐smokers. Figure 1 shows a flow chart of the search process.

### 3.2 Description of studies included

Table 2 gives a description of all the studies included. The mean sample size of the studies included was high, and it was noteworthy that in most of the studies, almost half the sample size corresponded to never‐smokers, and in four of them, all the participants were never‐smokers, meaning that the remaining 16 studies were not designed to assess the role of different risk factors in COPD in never‐smokers.

**TABLE 2:** Description of the studies included

Author and year	Type of study and country	Sample size	No. of never‐smokers (%)/no. of COPD never‐smokers (%)	No. of women (%)	COPD diagnosis	Risk factor analysed	Variable analysed	% increased risk of COPD/risk observed	Score
Orozco‐Levi et al,^20^ 2006	Case–control (hospital) Spain	120	94 (78.3%)/42 (70%)	120 (100%)	FEV1/FVC < 70% postBD	Biomass	Wood and charcoal	OR 4.5 (95%CI 1.4–14.2; *p* = 0.01)	5
Ekici et al,^21^ 2005	Case–control (population) Turkey	596	596 (100%)/140 (100%)	596 (100%)	FEV1/FVC < 70% preBD	Biomass	No. of hours cooking × no. of years: ‐Group A: 68.8 h‐years ‐Group B: 68.8–152.4 h‐years ‐Group C: >152.4 h‐years	23.1% (95% CI 13.4–33.2) ‐Group A: OR 1.7 (95%CI 1.0–3.1; *p* = 0.04) ‐Group B: OR 2.5 (95%CI 1.4–4.4; *p* = 0.001) ‐Group C: OR 3.3 (95%CI 1.9–5.7; *p* = 0.0001)	5
Regalado et al,^22^ 2006	Cross‐sectional Mexico	841	841 (100%)/111 (100%)	841 (100%)	FEV1/FVC < 70% preBD	Biomass	PM_10_ particulate concentration measured by nephelometer for 1 h while cooking	OR FEV1/FVC < 70% + FEV1 < 80%: 3.9 (95%CI 0.94–16.3; p = 0.06) OR FEV1/FVC < 70%: 1.3 (95%CI 0.7–2.4; *p* = 0.32) FEV1 decrease 81 mL or 4.7% of that predicted with PM_10_ > 2.6 mg/m^3^	4
Menezes et al,^19^ 2005	Cross‐sectional Brazil	999	428 (42.9%) never‐smokers/not registered	558 (55.8%)	FEV1/FVC < 70% postBD	Occupation Biomass	Occupation >10 years Exhibition charcoal stove for heating/cooking	Occupation increases COPD risk 36% (*p* = 0.08) Coal stove exposure increases COPD risk 40% (*p* = 0.08)	4
Tuner et al,^32^ 2012	Cohorts USA	811 961	375 087 (46.21%)/1797 deceased COPD never‐smokers	449 361 (54.5%)	COPD medical diagnosis	Radon	Radon measurement >100 Bq/m^3^	Mortality from COPD 1.03 (95%CI 0.86–1.25)	6
Lamprecht et al,^31^ 2011	Cross‐sectional China, Turkey, Austria, South Africa, Iceland, Germany, Poland, Norway, Canada, USA, Philippines, Australia, England, Sweden	10 000	4291 (42.9%)/523 (12.2%)	5231 (52.31%)	FEV1/FVC postBD <70%	Respiratory infections in childhood Occupation Asthma	Documented infections in childhood Occupation: Organic powder at least 10 years Asthma	Infections in childhood: OR females 2.21 (95%CI 0.89–5.47 *p* = 0.087) and OR males 2.82 (95%CI 0.94–8.51; *p* = 0.065) Occupation: OR females 1.96 (95%CI 1.2–3.2; *p* = 0.007), OR males 2.18 (95%CI 0.99–4.8; *p* = 0.054) Asthma: OR females 4.62 (95%CI 3.04–7.02; *p* < 0.001) and OR males 4.12 (95%CI 2.06–8.26; *p* < 0.001)	7
Perez‐Padilla et al,^29^ 2012	Cross‐sectional Latin America: Chile, Uruguay, Brazil, Venezuela, Mexico	5315	2278 (42.3%)/240 (10.5%)	3211 (60.41%)	FEV1/FVC < 70% postBD	Tuberculosis (TBC) Asthma	Previous diagnosis of tuberculosis or asthma	TBC: OR 3.66 (95%CI 1.4–9.55; *p* = 0.01) Asthma: OR 4.24 (95%CI 2.47–7.28; *p* < 0.001)	6
Lee et al,^30^ 2015	Cross‐sectional Korea	5784	3473 (57.7%)/258 (31.7%)	3053 (52.78%)	FEV1/FVC < 70% preBD	Occupation Tuberculosis	Occupation: manual labour: construction, mining Previous diagnosis of tuberculosis	7.6% Occupation: OR 2.6 (95%CI 1.3–5.3; *p* = 0.007) TBC: OR 4.5 (95%CI 2.3–8.7; *p* < 0.0001)	5
Yin et al,^24^ 2007	Cohorts China	6497	6497 (100%)/342	5957 (89.6%)	FEV1/FVC < 70% preBD	Passive smoking (home and workplace)	Density and duration (40 h/weeks for >5 years)	OR home: 1.60 (95%CI 1.23–2.10) OR workplace: 1.50 (95%CI 1.14–1.97; *p* = 0.002) OR home + workplace: 1.48 (95%CI 1.18–1.85 *p* = 0.001)	8
Zhou et al,^25^ 2009	Cross‐sectional China	20 245	12 471 (66.6%)/644 (38.6%)	10 236 (88.7%)	FEV1/FC < 70% postBD	Biomass Passive smoking	Exposure to biomass if used for cooking/heating more than 1 hour Passive smoking: exposure to tobacco at home/workplace	Biomass cooking: OR 1.31 (95%CI 1.08–1.58; *p* = 0.006) Biomass heating: OR 1.48 (95%CI 1.07–2.05; *p* = 0.017) Passive smoking: OR 1.31 (95%CI 1.06–1.61; *p* = 0.014)	8
Jordan,^26^ 2011	Cross‐sectional England	21 104	8859 (42%)/not registered	11 257 (53.3%)	FEV1/FVC < 70% preBD	Passive smoking	Exposure in hours/weeks	OR 1.98 (95%CI 1.03–3.79) if exposure greater than 20 h/week	5
Hagstad et al,^27^ 2014	Cross‐sectional Sweden	2118	2118 (100%)/140 (6.65%)	1251 (59.1%)	FEV1/FVC < 70% preBD	Passive smoking	Questionnaire on exposure to tobacco at home and in the workplace	OR previous home: 2.03 (95%CI 1.23–3.34; *p* = 0.005) OR all homes: 1.98 (95%CI 1.23–3.18; *p* = 0.005) OR previous workplace: 2.06 (95%CI 1.30–3.27; *p* = 0.002) OR all homes and workplaces 3.94 (95%CI 1.41–11; *p* = 0.009)	5
Hnizdo, ^13^ 2002	Cross‐sectional USA	9823	4369 (44.47%)/106 (15.29%)	5166 (52.59%)	FEV1/FVC < 70% preBD+FEV1 < 80%	Occupation	Occupation: industry and time	COPD fraction attributed to occupation in non‐smokers 31.1%	5
Blanc et al, ^14^ 2009	Cohorts USA	1504	323 (21.47%)/165 (13%)	876 (58.29%)	FEV1/FVC < 70% preBD	Occupation	Exposure to VGDF and JEM	OR 2 (95%CI 1.28–3.18)	7
Mehta et al,^15^ 2012	Cohorts Switzerland	4267	1940 (45%)/253 (43.84%)	2121 (49.7%)	FEV1/FVC < 70% preBD	Occupation	Biological powder Mineral powder Gases/vapours VGDF	IRR biological powder: 3.14 (95%CI 0.88–11.24) IRR mineral powder: 3.22 (95%CI 0.84–12.36) IRR gases/vapours: 3.94 (95%CI 1.23–12.58) IRR VGDF: 3.28 (95%CI 1.03–10.41)	7
Mahmood et al,^23^ 2017	Cross‐sectional India	200	113 (56.5%)/113 (56.5%)	Not registered	FEV1/FVC < 70% postBD	Biomass Tuberculosis Asthma	Questionnaire	Biomass: 53.98%, *p* = 0.0001 Tuberculosis: 32.74%, *p* = 0.0001 Asthma: 14.16%; *p* = 0.003	3
Hagstad et al,^16^ 2015	Cross‐sectional Sweden	1839	967 (52.6%)/74 (7.7%)	870 (47.3%)	FEV1/FVC < 70% postBD	Occupation	Exposure to gas, dust or vapours	OR 1.85 (95%CI 1.03–3‐33)	5
Tan et al,^28^ 2015	Cross‐sectional Canada	4893	2295 (47%)/147 (6.43%)	2797 (57.16%)	FEV1/FVC < 70% postBD	Asthma Childhood infections Passive smoking	Record of asthma or history of hospitalisation in childhood Passive smoking: partner of a smoker in the preceding 2 weeks	Asthma: OR 2.23 (1.36–3.66) for mild COPD, OR 4.94 (2.94–8.30) moderate–severe COPD Childhood infections: OR 4.80 (95%CI 2.43–9.46) for COPD moderate–severe Passive smoking: OR 2.60 (1.05–6.43) for mild COPD women	6
Ramadan,^17^ 2012	Cross‐sectional Egypt	300	120 (40%)/120 (40%)	70 (23.34%)	FEV1/FVC < 70% postBD	Occupation Biomass	Occupation: risk occupation more than 3 months Biomass: cooking exposure/heating for at least 6 months	Occupation: OR 1.09 (95%CI 0.41–0.78; *p* < 0.05) Biomass: OR 1.26 (95%CI 0.38–0.82; *p* < 0.05)	5
Denguezli et al,^18^ 2016	Cross‐sectional. Tunisia	661	485 (73.4%)/28 (4.7%)	352 (67.2%)	FEV1/FVC < 70% postBD	Occupation Biomass Asthma Childhood hospitalisation	Occupation: risk occupation at least 3 months Biomass: exposure at least 6 months Asthma diagnosis Childhood hospitalisation record	Occupation >10 years: OR 1.87 (95%CI 1140–12.863; *p* = 0.015) Biomass ≥ 10 years kitchen: OR 1.479 (95%CI 0.3–5.855; *p* = 0.578) Asthma: OR 10.621 (95%CI 2.897–38.937; *p* < 0.01) Childhood hospitalisation: OR 3.075 (95%CI 0.35–27.017; *p* = 0.311)	4

Abbreviations: JEM, job‐exposure matrices; postBD, post‐bronchodilator; preBD, pre‐bronchodilator; TBC, tuberculosis; VGDF, occupational exposure to vapours, gases, dust or fumes.

Table 3 summarises the main findings by the risk factor analysed. The total sample size of the included studies was 909 067, with a median of never‐smokers of 2029 (range 374 993) and a median of COPD never‐smoker of 143.5 (range 1769).

**TABLE 3:** Summary by risk factor analysed

Risk factor analysed	Number studies	OR (maximum)	OR (minimum)	Maximum score	Minimum score
Occupation	9	4.5	1.26	8	3
Biomass	8	3.94	1.09	7	4
Passive smoking	5	3.94	1.31	8	5
Asthma	5	10.621	4.24	7	3
Tuberculosis	3	4.5	3.66	6	3
Respiratory infections in childhood	3	4.80	3.075	7	4
Radon	1	1.03		6	

An individual analysis of each of the risk factors now follows.

### 3.3 Occupation

There were nine studies that analysed occupational exposure as a risk factor for development of COPD in never‐smokers. Of these, two were conducted in the United States^13^, ^14^: The first study^13^ found a fraction of COPD attributable to work in never‐smokers of 31.1%; and the second^14^ showed an increase in risk (OR 1.98, 95%CI 1.26–3.09) with occupational exposure to vapours, gases, dust or fumes (VGDF) and with job‐exposure matrices. A study conducted in Switzerland^15^ found evidence of an increased risk of COPD in never‐smokers with occupational exposure to biological powder (OR 3.14, 95%CI 0.88–11.24), mineral powder (OR 3.22, 95%CI 0.84–12.36), exposure to gas and vapours (OR 3.94, 95%CI 1.23–12.58) and VGDF (OR 3.28, 95%CI 1.03–10.41). Another study undertaken in Sweden^16^ obtained an OR of 1.85 (95%CI 1.03–3.33) for development of COPD with exposure to gas, dust or vapours. Two studies^17^, ^18^ showed a significant association between development of COPD in never‐smokers and holding a risk occupation for at least 3 months. Lastly, a study undertaken in Brazil^19^ established that occupation increases the risk of developing COPD by 36% (*p* = 0.08).

### 3.4 Biomass

There were eight studies included that analysed biomass as a risk factor for development of COPD. Three of these exclusively included women never‐smokers and analysed the effect of biomass as a risk factor for development of COPD.^20^, ^21^, ^22^ A Spanish study^20^ concluded that the combined use of wood and coal significantly raised the risk of COPD (OR 4.5, 95%CI 1.4–14.2). A study conducted in Turkey^21^ found that 23.1% of COPD cases could be attributed to exposure to biomass, with risk rising in response to greater exposure measured in hours per year. In Mexico, Regalado et al^22^ concluded that women who use biomass for cooking have a reduction in pulmonary function as compared with those who cook with gas. The study conducted by Mahmood et al^23^ in India showed exposure to a biomass source as being the principal risk factor for developing COPD, with a 54% increase in risk (*p* = 0.0001).

### 3.5 Passive smoking

The review included five studies that analysed the association between passive smoking and development of COPD in never‐smokers. Two of these were undertaken in China.^24^, ^25^ The first was a cohort study that included 6497 non‐smokers, 342 of whom had a diagnosis of COPD. It found an association between passive smoking in the home and workplace and an OR of 1.48 (95%CI 1.18–1.85) for a high level of exposure consisting of 40 h per week over the course of at least 5 years. The second reported a prevalence of COPD in never‐smokers of 5.2%, and an association with exposure to passive smoking in the home and in the workplace, with an OR of 1.31 (95%CI 1.06–1.61). Jordan^26^ conducted a study in the United Kingdom with the aim of analysing passive smoking as a risk factor for COPD: Among never‐smokers, the risk was 1.98 (95%CI 1.03–3.79) for an exposure of more than 20 h per week. In Sweden, Hagstad et al^27^ found an association between COPD and exposure to passive smoking in the home and the workplace, with an OR of 3.94 (95%CI 1.41–11, *p* = 0.009). A study undertaken in Canada^28^ showed a prevalence of COPD in never‐smokers of 6.4% (27% of all COPD) and analysed passive smoking as a risk factor, reporting an OR of 2.6 (95%CI 1.05–6.43) for women with mild COPD.

### 3.6 Asthma

There were five studies that analysed asthma as a risk factor for development of COPD in never‐smokers, and all found a significant association. The strongest association was reported in a study conducted in Tunisia by Denguezli et al,^18^ with an OR of 10.62 (95%CI 2.90–38.94, *p* < 0.01). The study undertaken in India^23^ reflected a 14.16% increased risk of COPD in subjects with a previous diagnosis of asthma.

### 3.7 Tuberculosis

The review included three studies that analysed previous diagnosis of tuberculosis as a risk factor for development of COPD. Perez‐Padilla et al^29^ conducted a study in Latin America that included 2278 never‐smokers, 240 of whom presented with COPD and were never‐smokers, and observed that the OR for development of COPD with previous diagnosis of tuberculosis was 3.66 (95%CI 1.4–9.55). A cross‐sectional study undertaken in Korea,^30^ which included 258 COPD never‐smokers, found an OR of 4.5 (95%CI 2.3–8.7) in subjects who had a previous diagnosis of tuberculosis. Similarly, the third study, conducted in India,^23^ reported a 32.74% increased risk of COPD in subjects with a history of tuberculosis.

### 3.8 Respiratory infections

There were three studies that analysed the role of childhood respiratory infections in the development of COPD. Lamprecht et al^31^ included 523 never‐smokers with diagnosis of COPD and found evidence to show that, in those with a history of respiratory infections in childhood, the OR was 2.21 (95%CI 0.89–5.47) in women and 2.82 (95%CI 0.94–8.41) in men. A study carried out in Canada^28^ showed an OR for moderate‐to‐severe COPD of 4.8 (95%CI 2.43–9.46). Lastly, a study conducted in Tunisia by Denguezli et al^18^ reported a threefold higher risk of developing COPD (OR 3.075, 95%CI 0.35–27.02) in subjects with a record of hospitalisation due to childhood respiratory infections.

### 3.9 Radon

The review included one study that analysed the association between radon and COPD. Turner et al^32^ carried out a cohort study in the United States with a large sample size and observed an association between radon concentration > 100 Bq/m^3^ and COPD mortality, which, in the case of never‐smokers, was 1.03 (95%CI 0.86–1.25). Furthermore, they found evidence of a significant positive linear trend in COPD mortality with increasing categories of radon concentrations (*p* = 0.05).

### 3.10 Quality of studies included

The quality of the studies reviewed ranged from 3 to 8 points, with a mean score of 5.5 points.

## 4 DISCUSSION

The studies reviewed show a significant association between incidence of COPD in never‐smoker patients and occupational exposure, exposure to biomass, passive smoking and having previously suffered from asthma, tuberculosis or respiratory infections during childhood. In addition, they suggest that residential radon could increase COPD mortality, though more research is needed to confirm this finding. In most of the studies and for most of the exposures analysed, the association observed was statistically significant. It should be noted that, among the risk factors, the effect is most pronounced for previous respiratory diseases (asthma, tuberculosis) or having presented with respiratory infections compared with exposures of an occupational or environmental nature.

More than 2800 million persons commonly use biomass fuels for cooking.^33^ The percentage varies widely among countries and regions and ranges from 30% to 75% in rural areas^34^, ^35^, ^36^: For instance, in countries such as India, biomass fuels are used for cooking and heating in almost 90% of rural homes and a third of urban dwellings. Indeed, every year, over 1.5 million persons around the world die of pneumonia, chronic respiratory diseases and lung cancer, due to indoor air pollution caused by biomass fuel used in cooking.^37^ Biomass fuels account for 2.9% of all deaths worldwide and 3.7% of the total morbidity and mortality burden in developing countries.^34^ This systematic review included studies that analysed biomass used for cooking and heating as a risk factor for development of COPD in never‐smokers in different geographical areas (Mexico, Turkey, Brazil, China, India, Egypt and Spain) and found an increased risk of COPD of 4.5 in a case–control study undertaken in Spain.^20^ Furthermore, this risk is observed to increase with the number of hours engaged in cooking.

Some studies on occupations that entail exposure to toxic gases in the workplace,^38^ grain dust on farms^39^ and fumes and dust in factories, have observed a strong association with development of COPD.^40^ The fraction attributable to occupation‐related COPD ranges from 9% to 31%,^41^ but the real attributable risk is unclear due to the fact that the definition of COPD is not standardised in epidemiological studies, particularly those carried out in developing countries. The studies with a cross‐sectional design included in this systematic review describe an association between occupation and development of COPD in never‐smokers. Hnizdo^13^ estimates a work attributable fraction for development of COPD in never‐smokers of 31.1%, with the main occupations being associated with the transport, stocking and handling of materials used in processing and construction. Other studies having a better design but smaller sample size than the above, conducted in the United States^14^ and Switzerland,^15^ also analysed occupation as a risk factor for development of COPD. Blanc et al^14^ found that exposure to VGDF was associated with double the risk of COPD being developed by never‐smokers, and Mehta et al^15^ described how exposure by an adult Swiss population to biological powder, mineral powder, gas/vapours and VGDF was associated with COPD, at least to a moderate degree, with the highest risk being posed by exposure to gas and vapours (OR 3.94).

An association has been reported for passive smoking as a potential risk factor for development of respiratory diseases, and the studies reviewed show a uniform increase in risk. The biological mechanism would presumably be the same as that observed in active smokers, though the inflammatory component and time of induction might possibly be less for never‐smokers than for active smokers. One of the studies found a stronger association with a higher number of weekly hours of exposure to active smoking habit^26^ or in cases of simultaneous exposure in the home and in the workplace.^27^

It has been reported that chronic inflammation of the respiratory tract and chronic airflow obstruction in asthma sufferers could cause remodelling due to thickening and fibrosis of the airways^42^ and that this remodelling could be progressive and irreversible, giving rise to development of COPD. There are similar mechanisms between development of chronic asthma and COPD, with an increase in neutrophils, proteases and oxidative stress. Moreover, and especially in the case of developing countries, one should bear in mind that inappropriate treatment of chronic asthma or severe asthma without inhaled corticosteroids could contribute to development of COPD. A longitudinal study^43^ conducted a follow‐up across 15 years and found that subjects with self‐reported diagnosis of asthma presented with a greater decline in FEV1, something that could be related with a baseline reduction in FEV1 and an increase in impaired lung function that is characteristic of COPD.

Pulmonary tuberculosis is associated with chronic airflow obstruction during diagnosis, treatment and years after undergoing treatment.^44^, ^45^ The degree of airflow obstruction is linked to disease spread, and the prevalence of obstruction varies between 28% and 68% of patients with tuberculosis. Patients with pulmonary tuberculosis generally develop a maximum loss of lung function within the 6 months following diagnosis and stabilise at 18 months of completing the treatment.^46^, ^47^ The biological mechanism responsible for this chronic obstruction of the respiratory tract might be the fibrosis of the airways caused by tuberculosis, as well as the immune response to mycobacteria that may cause inflammation of the respiratory tract, likewise characteristic of COPD. Furthermore, the degree of bronchial obstruction is related to disease severity as measured by radiological extension.

Indoor air pollution by biomass fuel is a factor that influences the development of respiratory infections during childhood and is an important cause of childhood mortality in developing countries, particularly across Asia and Africa.^48^ The survivors of these respiratory infections could present with factors that might predispose them to COPD in adult life. Other factors such as poverty, low socio‐economic level and malnutrition could contribute to the increase in respiratory infections during childhood. Several studies^49^, ^50^, ^51^ have shown that, after controlling for confounding factors such as smoking habit, persons who suffered from respiratory infections during childhood displayed lower FEV1 and FVC values, suggesting poor lung development. Another possible hypothesis is that there may be genetic factors that predispose persons to respiratory infections during childhood, as well as a lower FEV1 in adult life, though this hypothesis may imply that the alteration in pulmonary growth might precede infection of the respiratory tract. Bacterial infection due to *Streptococcus pneumoniae* and *Haemophilus influenzae* is frequently cited as the aetiology of severe pneumonia in children^52^ so that the impact of such infections on the prevalence of COPD in developing countries is likely to be higher due to their inappropriate treatment. The effect of having suffered from asthma, tuberculosis or previous respiratory infections on development of COPD in never‐smokers is considerably greater than that of other exposures.

Radon is the most important source of ionising radiation of natural origin for human beings.^53^ When it is inhaled, the solid particles into which the gas decays are retained in the lungs and irradiate alpha particles to the cells lining the lungs, bringing about molecular changes and possibly damaging DNA. In 2020, Conde‐Sampayo^54^ conducted a systematic review of exposure to residential radon and COPD and reported a possible trend towards the existence of this association, though no definitive conclusion could be reached. This implies that the effect of radon on COPD is unknown. Apart from the Cancer Prevention Study II, which observed an association between radon and COPD mortality, there is another ecological study undertaken in Galicia, but not included in this review, which did find evidence of an association between radon concentration and hospital admissions due to COPD.^55^ Another recent study, albeit conducted on smokers, has reported that radon increases risk of COPD in smokers.^56^ In light of these findings, studies are called for to analyse this association.

This review has some strengths, the most important of which is having been based on the PRISMA guidelines and, by extension, having used a rigorous method. Furthermore, a specific quality assessment scale was developed, which indicates that the studies are of medium quality. External validity is also high, in that the studies reviewed were conducted in different geographical areas.

Conversely, this review also has some limitations. The main limitation lies in the heterogeneity of the methodology used by the different studies, something that made it impossible to perform a meta‐analysis, overall or individual, for any of the risk factors analysed. The studies included also displayed differences in the definition of COPD so that, in some studies, only airflow obstruction was considered, without taking the reported symptomatology into account. We have not formally measured the risk of bias, because this study is not a meta‐analysis, but bias may be present due to different reasons in the included study (retrospective design, accuracy on measuring different risk factors [i.e. passive smoking or exposure to VGDF]). A further limitation is the use of a non‐validated scale to assess the quality of the included studies, though similar scales have been used by our group in other systematic reviews.^57^, ^58^, ^59^

In conclusion, a sizeable proportion of patients with COPD are never‐smokers, and many risk factors are implicated in the disease's development, fundamentally exposure to biomass fuel, risk occupations, a history of asthma or tuberculosis and exposure to passive smoking. The available literature points to evidence of an association between exposure to residential radon and COPD mortality. The absence of a greater number of studies specifically conducted on never‐smokers is extremely noteworthy, particularly when smoking in the most developed countries is progressively decreasing and the percentage of COPD in never‐smokers will necessarily be gradually increasing. The need for more research on this topic is evident. These studies should exclusively include never‐smokers and assess all the potential risk factors of COPD. The sample size should be high, at least 500 participants with more than 200 COPD never‐smokers to obtain reliable effect estimations. Strategies should be drawn up by the authorities, which are designed to reduce the risk of development of COPD in never‐smokers through lowering the exposure to toxic substances at home. Similarly, there is a need for more studies on never‐smokers, so as to allow for the possible role played by exposure to residential radon to be elucidated.

## CONFLICT OF INTEREST

None.

## ETHICS STATEMENT

Due to the nature of this study, Ethics Committee approval was not required.

## AUTHOR CONTRIBUTIONS

ARR conceived the idea and designed the methodology and the inclusion and exclusion criteria. APS did the bibliographic search. CCP, CRG, RG and CRR extracted the data. All authors have read and provided intellectual input to the manuscript. All authors have approved the final version of the manuscript and take public responsibility of its content.

## Data Availability

Data sharing is not applicable to this article as no new data were created or analyzed in this study.
